# Optimized small object detection in low resolution infrared images using super resolution and attention based feature fusion

**DOI:** 10.1371/journal.pone.0328223

**Published:** 2025-07-18

**Authors:** Weilun Wang, Jian Xu, Ruopeng Zhang

**Affiliations:** Big Data and Internet of Things School, Vocational Institute of Engineering, Chongqing, China; Purdue University, UNITED STATES OF AMERICA

## Abstract

Infrared (IR) imaging is extensively applied in domains such as object detection, industrial monitoring, medical diagnostics, intelligent transportation due to its robustness in low-light, adverse weather, and complex environments. However, challenges such as low resolution, high noise, limited texture details, and restricted dynamic range hinder the performance of traditional object detection models. To address these limitations, this study proposes an optimized approach for small object detection in low-resolution IR images by integrating super-resolution reconstruction with an enhanced YOLOv8 model. A lightweight super-resolution network, LightweightSRNet, is designed to enhance low-resolution IR images into high-resolution ones, improving feature quality with minimal computational complexity. To handle complex backgrounds and scale variations, a Hybrid Global Multi-Head Attention (HG-MHA) mechanism is introduced, enhancing target focus and suppressing noise. An improved SC-BiFPN module is developed to integrate cross-layer feature interactions, boosting small object detection by fusing low-level and high-level features. Additionally, a lightweight C2f-Ghost-Sobel module is designed for efficient edge and detail extraction with reduced computational cost, ensuring real-time detection capabilities. Experimental results on the HIT-UAV dataset show significant performance improvements, with Recall rising from 70.23% to 80.51% and mAP from 77.48% to 83.32%, along with robust performance on other datasets, demonstrating the model’s effectiveness for real-world IR applications. The source code and datasets used in this study are available at: https://github.com/RuopengZhang/infrared-detection-code.

## 1 Introduction

Infrared images, as an important data source in the field of computer vision, have been widely applied in areas such as object detection, industrial monitoring, medical diagnostics, and intelligent transportation due to their stable performance in low-light, adverse weather, and complex environments [1,2]. Compared with visible light images, infrared images capture the thermal radiation information from the surface of objects, eliminating the need for external light sources. Even under nighttime or occlusion conditions, they provide reliable data support [3]. However, infrared images also face unique challenges and limitations. Firstly, infrared images lack detailed texture information, as their feature representation relies on the thermal radiation properties of targets, making traditional texture- and color-based feature extraction methods less applicable. Secondly, infrared images often have lower resolution due to limitations in sensor performance and hardware costs, leading to insufficient target features. Additionally, infrared imaging devices are susceptible to thermal noise and sensor noise, with limited dynamic range and low contrast between targets and backgrounds, further complicating object detection [4].

In recent years, deep learning methods, especially the YOLO series models (e.g., YOLOv4, YOLOv5, and the latest YOLOv8), have achieved remarkable progress in object detection. However, these models are primarily optimized for visible light images and exhibit limitations in infrared image detection, such as restricted feature extraction, inadequate adaptation to multi-scale feature fusion, high computational costs for embedded hardware environments, and insufficient capacity to handle complex application scenarios [5]. These limitations hinder their applicability in the domain of infrared image object detection.

To address these challenges, this paper proposes an infrared image super-resolution reconstruction and improved YOLOv8-based object detection method. The approach includes several key components: a lightweight super-resolution network, LightweightSRNet, which reconstructs low-resolution infrared images into high-resolution ones with low model complexity, enhancing image details and providing higher-quality input features for object detection; a hybrid global multi-head attention mechanism (HG-MHA) that adaptively fuses global and local features to tackle complex backgrounds and inconsistent object scales, improving attention to target regions while suppressing background noise; an improved SC-BiFPN module for efficient cross-layer information interaction, which directly fuses low-level detail information with high-level semantic features to enhance small object detection performance and minimize information loss during propagation; and a lightweight C2f-Ghost-Sobel feature extraction module that combines Sobel edge enhancement with Ghost convolution to optimize feature extraction efficiency, reduce model parameters and computational costs, and enhance the ability to capture edge and detail features, meeting real-time infrared image detection requirements.

Experimental results demonstrate that, compared to the original YOLOv8 model, the proposed method achieves significant performance improvements across multiple datasets. On the HIT-UAV dataset, by progressively introducing infrared super-resolution reconstruction, the hybrid attention mechanism, the SC-BiFPN fusion strategy, and the lightweight C2f-Ghost-Sobel module, the model’s recall improves from 70.23% to 80.51%, mAP increases from 77.48% to 83.32%, and parameters are only increased by 0.1M, with FLOPs controlled at 8.6 GFLOPs. The contributions of each module were validated through ablation experiments, particularly excelling in small object detection and complex background scenarios. In addition, experiments on the FLIR dataset further verified the generalizability of the proposed method, demonstrating strong adaptability and robustness, and proving the effectiveness of the improved model in various infrared scenarios.

The primary contributions of this paper can be summarized as follows:

- A LightweightSRNet network is proposed, effectively achieving super-resolution reconstruction of low-resolution infrared images, enhancing image details while maintaining low model complexity.
- A hybrid global multi-head attention mechanism (HG-MHA) is designed, which improves attention to target regions and noise suppression through adaptive fusion of global and local features.
- An improved SC-BiFPN feature fusion module is constructed, introducing cross-layer information interaction mechanisms to significantly enhance small object detection performance.
- A lightweight C2f-Ghost-Sobel feature extraction module is developed, significantly reducing computational costs while enhancing detail feature extraction.

Through these innovations, the proposed infrared image super-resolution reconstruction and improved YOLOv8-based method not only improves the accuracy of infrared object detection but also achieves efficient detection of small objects and complex scenes while maintaining lightweight design and computational efficiency. This demonstrates its potential for practical applications.

## 2 Related work

In recent years, rapid advancements in deep learning have driven breakthroughs in image super-resolution reconstruction, attention mechanism optimization, small object detection, and lightweight network design. The integration of these technologies has provided innovative solutions for complex scene-based object detection, particularly in infrared image processing. However, challenges such as low resolution, complex backgrounds, indistinct target features, and real-time requirements persist. This section reviews state-of-the-art research on infrared image super-resolution, attention mechanisms, small object detection, and lightweight networks, providing theoretical and technical foundations for this study.

### 2.1 Infrared image resolution reconstruction

Infrared images often suffer from low resolution and insufficient details, posing challenges for object detection. Super-resolution reconstruction techniques enhance spatial resolution and provide high-quality inputs for downstream tasks. GAN-based methods such as FEGAN enhance high-frequency texture details [6], while multi-scale collaboration GANs incorporate dual-channel structures to improve fault detection [7]. The dense residual network leverages memory mechanisms and Wasserstein loss for superior image quality [8]. Sparse representation-based methods enhance details via signal matching, outperforming interpolation techniques in infrared scenarios [9]. Lightweight networks like PCDN use progressive feature distillation to reduce parameters [10], and pseudo-texture transfer enhances detail recovery with visible images [11]. Multi-scale models improve feature expression, performing well in lab-acquired infrared data [12], while nighttime infrared super-resolution integrates Retinex preprocessing for enhanced low-light performance [13]. Physics-enhanced models optimize wide field-of-view scenarios and vibrational spectroscopy applications [14,15].

### 2.2 Attention mechanism

Attention mechanisms improve infrared object detection by focusing on key regions while suppressing background noise. For small object detection, Combined-Attention YOLO enhances high-resolution feature layers [16], multi-scale self-attention improves DyHead structure for infrared traffic scenarios [17], and Mamba module-enhanced attention suppresses background interference in aerial infrared images [18]. For complex backgrounds, FA-YOLO incorporates dilated convolutional block attention to reduce false positives [19], while MWIRGas-YOLO uses a global attention mechanism for gas leakage detection [20]. Multi-modal feature fusion integrates infrared and visible images, such as MAF-YOLO, which employs dual-attention modules for better semantic representation [21], and triplet attention optimization, which enhances power equipment detection [22]. For lightweight models, YOLO-FIRI optimizes efficiency by compressing channels and integrating attention modules [23], and an anchor-free detection head further improves speed and accuracy [24]. Attention mechanisms also excel in specialized infrared scenarios like hazardous gas detection [25].

### 2.3 Infrared small target detection

Infrared small object detection remains challenging due to low contrast, small sizes, and limited texture features. Feature extraction optimization includes an improved FCOS method integrating spatio-temporal features to suppress background noise [26], DI-U-Net combining high-resolution multi-layer structures for effective feature learning [27], and CBAMV2 strengthening spatial and semantic feature fusion for improved robustness [28]. Attention-enhanced detection includes a YOLOv8-based multi-level feature fusion model for small object focus [29], MBFormer-YOLO with adaptive feature fusion for efficiency [30], and Recursive Feature Pyramid improvements for low-contrast target detection [31]. Lightweight designs such as the IRSDT framework integrate full-image and cropped-image detection to reduce complexity [32], while quantization-based optimization lowers storage demands while maintaining accuracy [33]. Synthetic data augmentation also enhances small object detection, with GAN-generated target masks addressing data scarcity [34] and multi-scale feature fusion improving robustness in complex backgrounds [35]. In addition, more general-purpose research on feature fusion mechanisms also contributes to improving detection performance. Dai et al. proposed Attentional Feature Fusion, a unified attention-based feature fusion scheme applicable across layers and skip connections, which improves fusion of features with inconsistent semantics and scales through multi-level attention modules [36]. Furthermore, Deshpande introduced a thermal feature detection framework that treats thermal images as color representations and utilizes a triplet-based Siamese CNN to robustly extract discriminative local features, showing enhanced performance on texture-deficient thermal imagery [37].

### 2.4 Network lightweight

Deep learning models for infrared object detection often face challenges in resource-constrained environments due to high computational costs. Lightweight backbone networks such as MobileNet V2-based models balance tracking accuracy and inference speed [38], while Transformer-based low-resolution thermal face detection achieves 30 FPS real-time detection on Raspberry Pi [39]. Edge computing optimizations include Edge-YOLO, which replaces the YOLOv5m backbone with ShuffleBlock and strip depthwise convolution attention, reducing computational complexity by 70.3% while maintaining accuracy [40], and YOLOv8n with PConv and coordinate attention, which reduces model size by 34.17% while improving detection accuracy [41]. Structural optimizations such as YOLOv5-IRL with spatial and channel attention cut parameters by 45.6% while boosting mAP [42], and SSD-based lightweight models refine anchor box con

urations for small object detection with an average accuracy of 80% [43]. Further optimizations include enhanced YOLOv5 architectures and activation functions for space infrared sensor applications [44], MSIA-Net with asymmetric convolution and lightweight fusion to reduce information loss [45], and RT-DETR modular optimizations for efficiency in constrained environments [46]. Lastly, YOLO-SGF integrates cross-scale feature fusion and custom loss functions, improving detection in complex infrared backgrounds while reducing computational costs [47].

## 3 Method

### 3.1 LightweightSRNet

**Fig 1:**
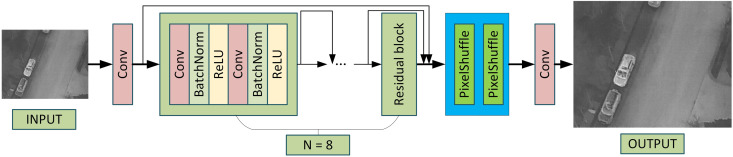
Framework of LightweightSRNet.

To address the low-resolution issue in infrared imaging and enhance image details, this study proposes a lightweight super-resolution network, LightweightSRNet, as illustrated in Fig 1, designed to achieve efficient and high-quality infrared image super-resolution reconstruction. By leveraging a streamlined design of residual blocks and employing a PixelShuffle upscaling strategy, the network effectively reduces model complexity while maintaining superior reconstruction performance. The overall structure and workflow are described as Fig 1.

The complete reconstruction process is as follows: the low-resolution infrared image is first processed by an initial convolution layer to extract basic features. These features are then passed through eight residual blocks to capture deep global and local features. Subsequently, two PixelShuffle layers progressively upscale the feature maps to generate a high-resolution infrared image. Finally, the high-resolution output is fed into an improved object detection network for further target detection tasks.

The LightweightSRNet features an optimized design with only eight key residual blocks for feature extraction. Each residual block consists of standard convolution layers and skip connections, which can be mathematically expressed as:


$$F_{residual}(x)=W_{2}\cdot ReLU(W_{1}\cdot x+b_{1})+b_{2}$$ (1)

where $W_{1}$ and $W_{2}$ denote the weight matrices of the convolutional layers, $b_{1}$ and $b_{2}$ are bias terms, x is the input feature map, and ReLU is the activation function. This structure preserves essential input information through skip connections, reducing computational costs without sacrificing reconstruction accuracy.

To further enhance resolution while preserving detail, the network employs two PixelShuffle layers to progressively upscale feature maps to the target resolution. The PixelShuffle operation, which rearranges channel dimensions for spatial reconstruction, is mathematically represented as:


$$I_{HR}=PS(F_{LR})$$ (2)

where $I_{HR}$ is the high-resolution output image, $F_{LR}$ is the low-resolution input feature, and PS denotes the PixelShuffle operation.

The core concept of PixelShuffle is to rearrange the channel dimensions of the input feature map into higher spatial resolution information. Assume the input low-resolution feature map $F_{LR}$ has dimensions of $\left(C\times r^{2},H,W\right)$, where C represents the number of input channels, r is the upscaling factor, and H,W denote the height and width of the feature map, respectively.

The goal of PixelShuffle is to transform $F_{LR}$ into a high-resolution feature map $I_{HR}$ with dimensions (C,rH,rW). The rearrangement process can be expressed as:


$$I_{HR}(c,i,j)=F_{LR}(c\times r^{2}+\left\lfloor i/r\right\rfloor \times r+(jmodr),\left\lfloor i/r\right\rfloor ,\left\lfloor j/r\right\rfloor )$$ (3)

where c=0,1,...,C−1 represents the index of the output channels,i,j denotes the pixel position in the high-resolution feature map, and r is the upscaling factor, which specifies the scaling ratio in the spatial dimensions.

In other words, PixelShuffle maps $C\times r^{2}$ input channels into C output channels while increasing the spatial resolution from (H,W) to (rH,rW). This operation reallocates features pixel-by-pixel into higher spatial resolution, effectively avoiding the artifacts commonly associated with traditional transposed convolutions.

### 3.2 Improved YOLOv8 model

This study is based on the YOLOv8 object detection framework. By introducing a hybrid attention mechanism, an improved feature fusion module, and lightweight convolution combined with an edge detection module, the performance of infrared image object detection is significantly enhanced. YOLOv8 employs an improved backbone network based on CSPNet, leveraging a dynamic anchor box mechanism and an efficient loss function to achieve outstanding object detection performance.

The basic structure of YOLOv8 comprises three main components: the Backbone, Neck, and Head. The Backbone is responsible for extracting multi-level features from the input image, preserving critical semantic information while reducing resolution. The Neck uses a feature pyramid structure to fuse multi-scale features, enhancing adaptability to small objects and complex scenes. The Head predicts the object classes and bounding box information based on the fused features.

**Fig 2:**
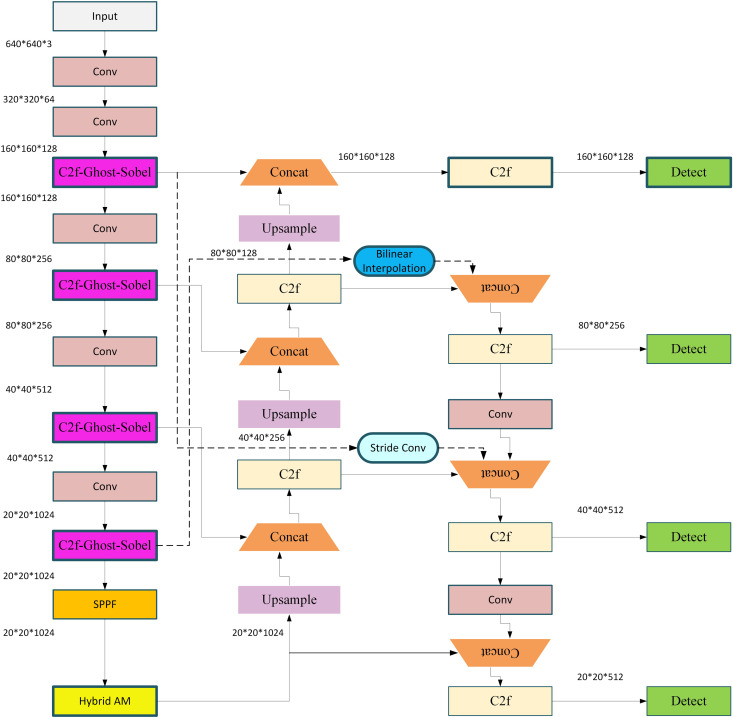
Overall architecture of the improved YOLOv8 network.

Despite YOLOv8’s remarkable performance in terms of speed and accuracy, its effectiveness in detecting low-resolution infrared images still has limitations. To address this shortcoming, we optimized its structure, with the improved overall architecture illustrated in Fig 2. The components marked with bold boxes represent the modifications made to the original YOLOv8 network. The small object layer is an existing feature provided by the original network, which is merely adopted in this work and will not be elaborated upon. Therefore, the subsequent focus will be on describing the other three improvements tailored for the characteristics of infrared images.

#### 3.2.1. Hybrid global multi-head attention mechanism.

**Fig 3:**
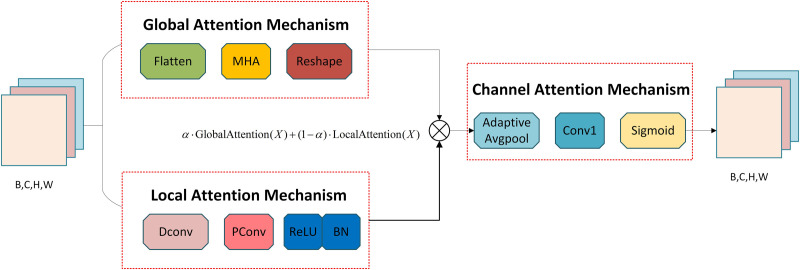
Structure of the HG-MHA.

The Hybrid Global Multi-Head Attention Mechanism (HG-MHA) aims to address the challenges in infrared image object detection, including complex backgrounds, blurry boundaries, and inconsistent target scales. As shown in Fig 3, by integrating global multi-head attention, local attention, and channel attention, this mechanism effectively captures both global and local information during feature extraction.

Global multi-head attention captures global contextual information by modeling long-range dependencies, enhancing the network’s ability to perceive target regions. Specifically, the input feature map X is linearly transformed to generate query (Q), key (K), and value (V), which are weighted using attention weights $softmax\;(\frac{QK^{T}}{\sqrt{d_{k}}})$ to produce global feature representations. The formula is as:


$$GlobalAttention(Q,K,V)=softmax\left(\frac{QK^{T}}{\sqrt{d_{k}}}\right)V$$ (4)

where $d_{k}$ represents the dimension of the key vector.

Local attention efficiently extracts detailed features through depthwise and pointwise convolution operations, focusing on local textures and boundary information of the target. Its mathematical representation is:


$$LocalAttention(X)=X*W_{local}$$ (5)

where X is the input feature map, and $W_{local}$ is the local convolution kernel. This mechanism preserves more target-relevant local details while significantly reducing computational complexity.

Channel attention dynamically reweights the feature channels, further enhancing the network’s focus on critical features associated with the target. The implementation of channel attention involves global average pooling (GAP) and two fully connected layers, as described by the following formula(6):


$$ChannelAttention(F)=\sigma (W_{2}ReLU(W_{1}GAP(F)))$$ (6)

where $W_{1}$ and $W_{2}$ are learnable parameters, and σ represents the Sigmoid activation function.

To efficiently combine global and local features, the mechanism introduces a multi-scale feature fusion strategy. By leveraging a learnable fusion parameter σ, global features $F_{global}$ and local features $F_{local}$ are weighted and fused as formula(7):


$$F_{out}=\alpha F_{global}+(1-\alpha )F_{local}$$ (7)

This strategy balances global semantic information with local detail features, effectively improving the model’s adaptability to small targets and complex backgrounds.

The overall design ensures that the attention mechanism effectively emphasizes target regions in infrared image object detection while suppressing background interference, providing more discriminative feature representations for subsequent detection networks.

#### 3.2.2. Improved feature fusion module.

To improve multi-scale feature fusion in infrared image object detection, this study proposes an enhanced feature fusion module, SC-BiFPN (Sparse Cross-layer Bidirectional Feature Pyramid Network), built on the BiFPN framework. The design introduces key cross-layer connections to better facilitate the interaction between high-level semantic information and low-level detail features, optimizing small object detection.

**Fig 4:**
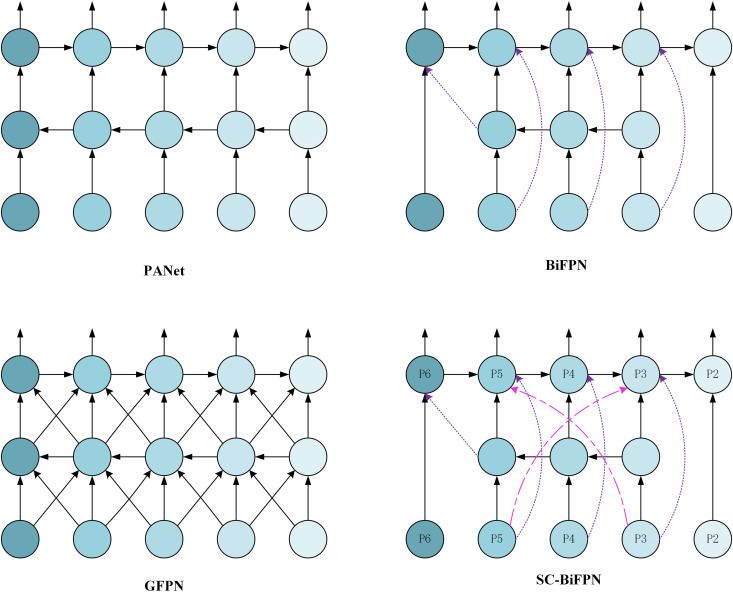
Comparison of SC-BiFPN with other fusion methods.

As shown in Fig 4, BiFPN excels in efficient bidirectional feature propagation; however, it lacks direct cross-layer interaction [48]. This limitation often leads to the loss of critical detail features during intermediate layer propagation, especially for small objects in infrared images. On the other hand, GFPN (Generalized Feature Pyramid Network) employs fully connected paths, which enhance information flow but also introduce redundant computation and high overhead [49]. SC-BiFPN is designed to combine the efficiency of BiFPN with the flexibility of GFPN by adding a limited number of key cross-layer connections, significantly improving the detection of small objects.

SC-BiFPN preserves BiFPN’s core bidirectional pathways, enabling both top-down and bottom-up feature transmission. This ensures the comprehensive capture of multi-scale target features in infrared images. To address feature propagation challenges in infrared small object detection, SC-BiFPN incorporates critical cross-layer connections, including low-to-high layer connection (P3 → P5) for directly passing low-level detail features to higher levels and high-to-low layer connection (P5 → P3) for guiding low-level feature optimization with high-level semantic information. These connections enhance the semantic modeling capabilities and improve object localization.

The cross-layer connections in SC-BiFPN are implemented with lightweight modules. For the upsampling pathway, bilinear interpolation is used to expand the feature map resolution, followed by a 1 × 1 convolution to align channel dimensions. The process is described by the formula(8):


$$F_{up}=Conv_{1\times 1}(UpSample(F_{in},s))$$ (8)

where $F_{in}$ is the input feature map, $F_{up}$ is the upsampled feature map, s represents the upsampling scale, and $Conv_{1\times 1}$ performs channel alignment.

For the downsampling pathway, strided convolution is used for direct feature map downsampling, with Batch Normalization (BN) applied to stabilize training. The process is described by:


$$F_{down}=BN(Conv_{3\times 3}(F_{in},stride=2))$$ (9)

where $F_{down}$ is the downsampled feature map.

The final fusion mechanism combines features from bidirectional pathways and cross-layer connections. The output feature is computed as:


$$F_{out}=\alpha F_{up}+\beta F_{down}+\gamma F_{cross}$$ (10)

where $F_{up}$, $F_{down}$, and $F_{cross}$ denote features from the upsampling path, downsampling path, and cross-layer connections, respectively. The learnable weights α, β, and γ dynamically adjust the contributions of different features.

This enhanced fusion strategy ensures that SC-BiFPN effectively balances global semantics and local details, providing a robust framework for detecting small objects in infrared images. Its lightweight design further ensures computational efficiency, making it suitable for deployment in resource-constrained environments while maintaining high performance.

#### 3.2.3. Lightweight C2f-Ghost-Sobel module.

To enhance edge detail sensitivity while maintaining computational efficiency, this study introduces the lightweight C2f-Ghost-Sobel module. Designed specifically for infrared image object detection tasks, this module integrates Ghost Convolution for parameter reduction, Sobel edge enhancement for global edge feature extraction, and a shortcut branch to preserve feature flow and gradient propagation. The overall structure achieves a balance between lightweight design and enhanced feature representation.

• Ghost Convolution for Lightweight Design

**Fig 5:**
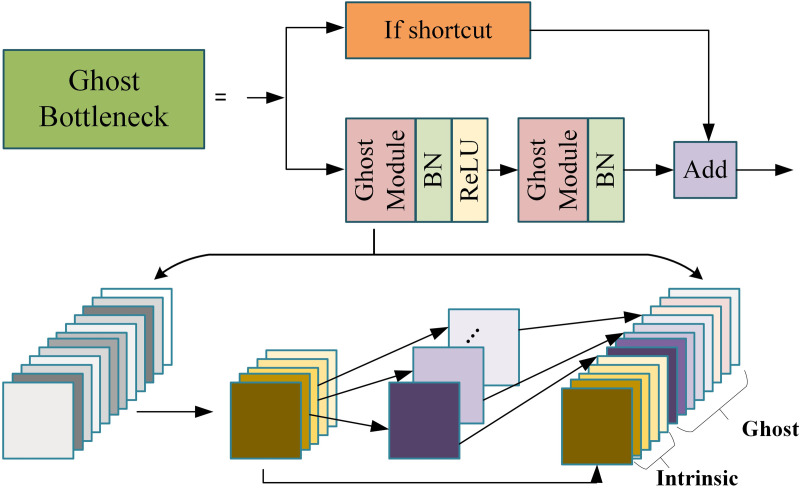
Schematic Diagram of the Ghost Bottleneck Structure.


As shown in Fig 5, To reduce computational cost, all convolution operations in the Bottleneck layers are replaced with Ghost Convolutions. This approach generates primary features and leverages simple linear operations to create redundant features, effectively reducing parameters and FLOPs while preserving feature representation. The mathematical formulation of Ghost Convolution is as:

$$F_{ghost}=F_{primary}+L\left(F_{primary}\right)$$ (11)

where $F_{primary}$ represents the features obtained from the primary convolution, and L(⬝) denotes the linear transformation applied to generate redundant features.

• Sobel edge enhancement for global edge features

After the Bottleneck layers, the Sobel operator is applied to enhance global edge features, emphasizing boundary details critical for detecting small and indistinct targets in infrared images. The Sobel edge gradient is computed using the horizontal ($K_{x}$) and vertical ($K_{y}$) convolution kernels:


$$K_{x}=\left[\begin{array}{ccc} {}*20c-1 & 0 & +1\\ -2 & 0 & +2\\ -1 & 0 & +1 \end{array}\right],\;K_{y}=\left[\begin{array}{ccc} {}*20c+1 & +2 & +1\\ 0 & 0 & 0\\ -1 & -2 & -1 \end{array}\right]$$

The gradients in the horizontal and vertical directions are computed as:


$$G_{x}=I(x,y)*K_{x},\;G_{y}=I(x,y)*K_{y}$$ (12)

where I(x,y) represents a given input image, *represents the convolution operation.

The magnitude of the edge gradient is then obtained as:


$$G=\sqrt{G_{x}^{2}+G_{y}^{2}}$$ (13)

To simplify computation, an absolute approximation is often used:


$$G\approx |G_{x}|+|G_{y}|$$ (14)

• Feature fusion with shortcut connections

The module fuses features from three branches: the shortcut branch, the main Bottleneck branch, and the Sobel-enhanced edge features. These are combined through concatenation, followed by a 1 × 1 convolution to reduce dimensionality:


$$F_{out}=Conv_{1\times 1}\left(Concat\left(F_{shortcut},F_{main},F_{edge}\right)\right)$$ (15)

where $F_{shortcut}$, $F_{main}$ and $F_{edge}$ are the shortcut features, main branch features, and edge-enhanced features, respectively.

**Fig 6:**
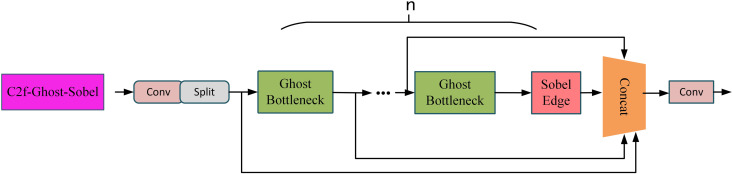
Structural Workflow of the C2f-Ghost-Sobel Module.

The structural workflow provides a detailed outline of the modular design and integration process for the proposed method, leading seamlessly into its specific description as Fig 6.

The structural workflow of the C2f-Ghost-Sobel Module involves several stages. Initially, the input features ($F_{in}$) are divided into two branches: a shortcut branch, which preserves shallow features ($F_{shortcut}$), and a main branch, where deep features are processed through n stacked lightweight Bottleneck layers utilizing Ghost Convolutions to extract hierarchical features ($F_{main}$). After feature extraction in the Bottleneck layers, a Sobel operator is applied globally to enhance the edge features, resulting in $F_{edge}$. Finally, the features from the shortcut branch, main branch, and Sobel edge enhancement are concatenated and reduced via a 1 × 1 convolution to generate the final output ($F_{out}$).

## 4. Experiments and results

This section begins with a detailed introduction to the datasets used in this study, including their sources, characteristics, and preprocessing methods. Next, the experimental setup and training strategies are described, covering hardware configurations, training parameter settings, and optimization details of the model. Subsequently, the evaluation metrics used to assess model performance are elaborated to ensure a comprehensive and objective analysis of the experimental results. Comparative experiments with state-of-the-art object detection models are conducted to validate the effectiveness of the proposed method. Using YOLOv8 as the baseline model, the improvements achieved in terms of accuracy, recall, and mAP are highlighted. Additionally, to further verify the applicability of the proposed approach, extended experiments were performed on other datasets of varying types. The results demonstrate the proposed method’s superior performance and robustness across diverse scenarios and tasks.

### 4.1. Datasets

This study primarily utilizes the HIT-UAV dataset [50] for model training and performance evaluation, and employs the **FLIR** [51]**, KAIST** [52]**, and DroneVehicle** [53] datasets as auxiliary validation sets to verify the algorithm’s generalization and adaptability.

**Fig 7:**
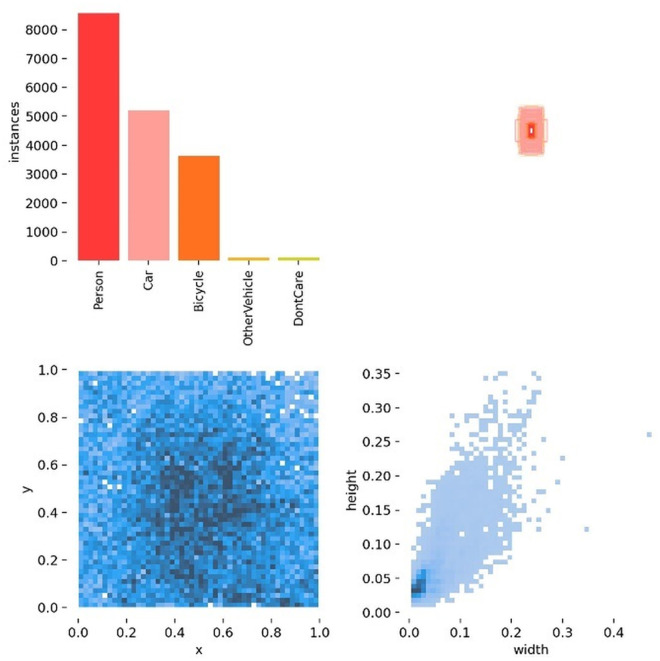
Target Distribution in the HIT-UAV Dataset.

The HIT-UAV (High-altitude Infrared Thermal dataset for Unmanned Aerial Vehicle-based object detection) is specifically designed for high-altitude infrared thermal imaging object detection with UAVs. This dataset contains 2,898 high-quality infrared thermal images extracted from 43,470 frames of video captured by UAVs in diverse environments, including schools, parking lots, roads, and playgrounds. It encompasses common object types such as pedestrians, bicycles, cars, and other vehicles, while also documenting variations in UAV flight altitudes (ranging from 60 meters to 130 meters) and camera angles (from 30 degrees to 90 degrees). These characteristics increase the dataset’s complexity and broaden its application scope. Additionally, the HIT-UAV dataset covers both daytime and nighttime lighting conditions, demonstrating the robustness of infrared imaging systems under complex lighting environments. This diversity makes the HIT-UAV dataset particularly effective for evaluating the performance of high-altitude infrared object detection algorithms, especially in multi-object and complex scenes. To validate the proposed algorithm, the original images in the dataset were downsampled to create low-resolution infrared images, with the original images serving as ground truth labels. The dataset was divided into training, validation, and testing sets in an 8:1:1 ratio. As shown in Fig 7, a high proportion of small targets is evident, making the dataset particularly suitable for evaluating the detection performance of small objects.

To further validate the algorithm’s generalization and adaptability, this study also employs the FLIR, **KAIST**, and **DroneVehicle** dataset as auxiliary validation sets. The FLIR dataset is a publicly available infrared thermal imaging dataset widely used for research and validation in object detection tasks. It contains infrared thermal images captured under various typical scenarios, covering both daytime and nighttime conditions. The primary object types include pedestrians and vehicles. Unlike the HIT-UAV dataset, which focuses on high-altitude scenarios, the FLIR dataset primarily targets infrared object detection in lower-altitude scenes. This difference in scenarios provides an additional experimental dimension, enabling the performance evaluation of the model to encompass a broader range of application scenarios. The KAIST Multispectral Pedestrian Detection dataset is a benchmark dataset composed of aligned visible and thermal image pairs captured in urban driving environments. It covers various illumination conditions and provides rich pedestrian annotations over 95,000 frames, making it particularly suitable for validating detection performance under real-world multispectral settings. The DroneVehicle dataset focuses on aerial vehicle detection tasks using infrared imagery. It includes numerous vehicle types observed from varying UAV perspectives, and presents challenges such as scale variation, dense traffic, and partial occlusion, making it an effective supplement for evaluating the robustness of the proposed model in dynamic traffic scenarios.

### 4.2. Experimental environment and parameters

In this study, YOLOv8n was selected as the baseline model for research and improvement. The model training was conducted on a Windows 10 system, utilizing an NVIDIA Quadro RTX 8000 GPU and an Intel Xeon Gold 5220R CPU. The software environment included Python 3.10.12 and PyTorch 2.4.1 with CUDA 11.8 support. The training process spanned 200 epochs, with a batch size of 16 and 8 worker threads to enhance data loading efficiency. Input image dimensions were set at 640 × 640 pixels, and Mosaic data augmentation was disabled (close_mosaic = 0). The Adam optimizer was employed to ensure stable convergence, with the initial learning rate (lr0), learning rate factor (lrf), and learning rate momentum set to 0.01, 0.2, and 0.937, respectively. These training configurations were designed to balance computational resources and model performance, ensuring effective generalization within a reasonable training time.

### 4.3. Evaluation metrics

This study employs Precision (P), Recall (R), and mean Average Precision (mAP) to evaluate the model’s detection accuracy, with the mAP threshold set to 0.5. Additionally, parameter count is used to measure the computational scale of the model, while the model’s weight size serves as an indicator of its deployability. The calculation formulas for Precision and Recall are given as follows:


$$P=\frac{TP}{TP+FP}$$ (16)

$$R=\frac{TP}{TP+FN}$$ (17)

where TP represents the number of correctly predicted positive samples, FP indicates the number of incorrectly predicted negative samples, and FN refers to the number of incorrectly predicted positive samples.

The Average Precision (AP) is defined as the mean of precision values across different recall levels for a given IoU threshold. The mAP is obtained by averaging the AP values over all classes. The calculation formulas are expressed as:


$$AP=\int_{0}^{1}\;P(R,dR$$ (18)

$$mAP=\frac{1}{N}\sum_{N}^{i=1}AP_{i}$$ (19)

where N is the total number of classes. These metrics ensure a comprehensive evaluation of the model’s accuracy, computational efficiency, and deployment feasibility.

### 4.4. Experiment results

A series of experiments were conducted to comprehensively evaluate the performance of the ISR-YOLOv8 model. First, we compared the target detection performance of YOLOv8 on low-resolution infrared images and infrared super-resolution reconstruction methods (including Bicubic interpolation, SRCNN, and our proposed LightweightSRNet). Next, we assessed the impact of different attention mechanisms on detection performance and conducted ablation studies to validate the contribution of each improvement module.

Furthermore, ISR-YOLOv8 was compared with widely used object detection models, including Faster R-CNN [54], SSD [55], YOLOv5s, YOLOv7 [56], **YOLO-IR-Free** [24] **and YOLO-DeepOC-IR** [57] to comprehensively demonstrate its advantages. Finally, the model was validated on two datasets, providing further evidence of ISR-YOLOv8’s effectiveness and adaptability for infrared image object detection tasks.

#### 4.4.1. Comparison of different reconstruction methods.

**Fig 8:**
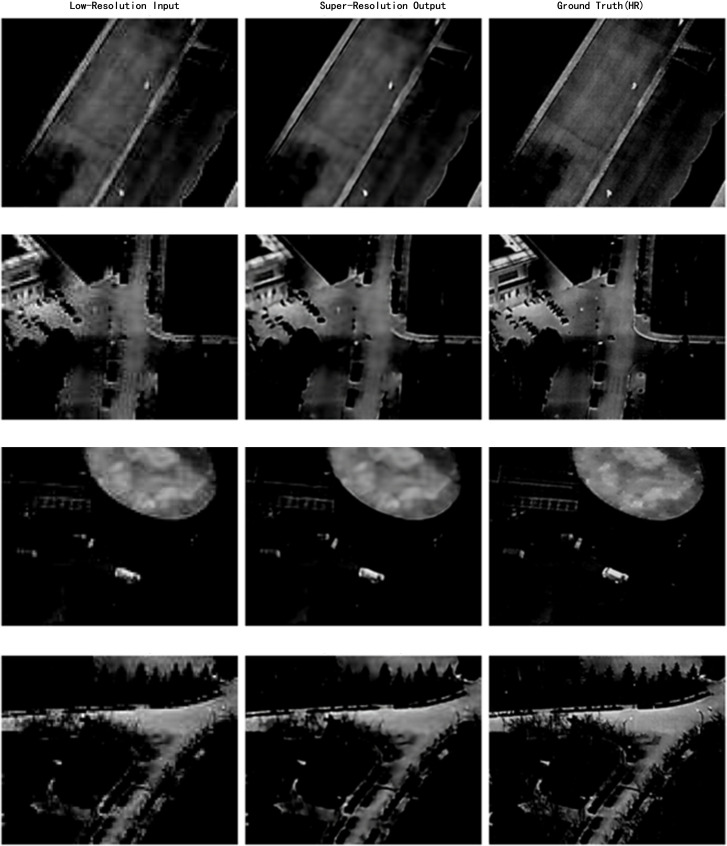
Comparison of Images Before and After Super-Resolution Reconstruction. (a) Before Reconstruction; (b) After Reconstruction; (c) GT.

A series of experiments were conducted to evaluate the target detection performance of YOLOv8 combined with low-resolution infrared images and different reconstruction methods, including Bicubic [58], SRCNN [59], and LightweightSRNet. The comparisons between reconstructed images and ground truth (GT) are illustrated in Fig 8.

The detailed results are shown in the Table 1.

**Table 1:** Comparison of detection performance using different RS methods.

Method	Recall	Precision	mAP	Params	FLOPs
Low-Resolution Infrared + YOLOv8	70.23%	82.16%	77.48%		
Bicubic+yolov8	73.20%	83.50%	79.10%	3.8M	1.2
SRCNN+yolov8	75.05%	83.98%	80.20%	4.0M	1.5
LightweightSRNet+yolov8	77.55%	84.43%	81.07%	3.5M	1.0

The results demonstrate that LightweightSRNet achieves excellent performance in balancing model efficiency and accuracy. Compared with Bicubic and SRCNN, LightweightSRNet significantly reduces parameter count (3.5M) and FLOPs (1.0) while achieving superior detection performance. Specifically, Bicubic, as a simple interpolation-based method, tends to produce blurred edges, which affects detail preservation. Although SRCNN employs multiple convolutional layers to enhance feature extraction, it suffers from larger model size (4.0M parameters and 1.5 FLOPs), limiting its ability to improve edge details and small-object detection.

The improved design of LightweightSRNet integrates a reduced number of residual blocks and a step-by-step upscaling strategy using PixelShuffle to enhance feature extraction and ensure high-resolution image generation. The streamlined residual blocks focus on extracting key features, avoiding redundant computations, while the PixelShuffle technique minimizes artifacts and maintains high output quality. Experimental results show that LightweightSRNet achieves significant improvements in mAP (81.07%), Recall (77.55%), and Precision (84.43%) compared to low-resolution images, outperforming both Bicubic and SRCNN. These results validate its effectiveness and efficiency for infrared object detection tasks.

#### 4.4.2. Comparison of different attention mechanisms.

To evaluate the performance of the Hybrid Global Multi-Head Attention (HG-MHA) mechanism, this study compares it with commonly used attention mechanisms (CBAM [60], CA [61], and SE [62]). The experimental results are summarized in the Table 2.

**Table 2:** Comparison of different attention mechanisms in infrared object detection.

Method	Recall	Precision	mAP	Params	FLOPs
LightweightSRNet+yolov8	77.55%	84.43%	81.07%	3.1M	8.2
LightweightSRNet+yolov8 + CBAM	78.63%	83.12%	82.14%	3.3M	8.8
LightweightSRNet+yolov8 + CA	78.12%	83.58%	82.32%	3.2M	8.7
LightweightSRNet+yolov8 + SE	77.89%	84.10%	82.08%	3.2M	8.6

From the table, it can be observed that incorporating different attention mechanisms generally improves Recall and mAP. HG-MHA achieves the best performance, with Recall increasing to 79.74% and mAP reaching 83.16%, representing improvements of 2.19% and 2.09%, respectively, over the baseline model (Resolution Reconstruction + YOLOv8). Compared to other attention mechanisms, HG-MHA shows clear advantages in Recall and mAP, though Precision slightly decreases from 84.43% to 83.81%. This suggests that HG-MHA prioritizes improving Recall, which is particularly beneficial for detecting multiple targets in complex scenarios.

In contrast, CBAM and CA exhibit similar performance, with mAP values of 82.14% and 82.32%, respectively, both significantly higher than the baseline but slightly lower than HG-MHA. The SE mechanism achieves a relatively high Precision (84.10%) but shows smaller improvements in Recall (77.89%) and mAP (82.08%).

Notably, while HG-MHA introduces additional computational costs (FLOPs increased to 9.3), its parameter count remains controlled at 3.4M, ensuring a lightweight design suitable for embedded devices and practical applications. These results demonstrate that HG-MHA achieves superior detection accuracy and Recall at a reasonable computational cost, making it particularly effective for small target detection and multi-object scenarios in complex infrared environments.

#### 4.4.3. Ablation experiments.

To verify the specific contribution of each improvement module to the performance enhancement of the model, multiple ablation experiments were conducted. The experimental results are shown in Table 3.

**Table 3:** Results of ablation experiments.

Method	Recall	Precision	mAP	Params	FLOPs
Low-resolution + YOLOv8	70.23%	82.16%	77.48%	3.1M	8.2
LightweightSRNet +YOLOv8	77.55%	84.43%	81.07%	3.1M	8.2
+ HG-MHA	79.74%	83.81%	83.16%	3.4M	9.3
+ SC-BiFPN	80.27%	83.58%	83.35%	3.5M	9.5
+ C2f-Ghost-Sobel	80.51%	83.01%	83.32%	3.2M	8.6

**Fig 9:**
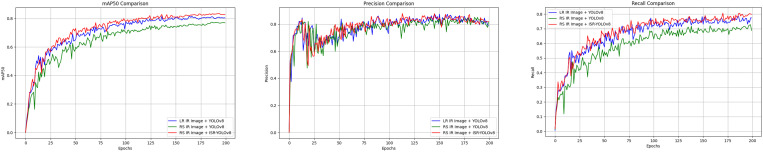
Comparison of Training Results.

The comparison of training results among Low-resolution + (YOLOv8), LightweightSRNet + (YOLOv8), and the three improved YOLOv8 methods is shown in Fig 9.

• Overall analysis

Through Fig 9, it can be visually observed that the introduction of resolution reconstruction (LightweightSRNet) significantly enhances the model’s detection capability. Specifically, the model with resolution reconstruction demonstrates notable advantages in Recall and mAP. Recall increased from 70.23% in low-resolution images to 77.55%, indicating that the model can capture more small and edge targets after resolution reconstruction, thereby reducing missed detections. Meanwhile, mAP improved from 77.48% to 81.07%, proving that the model achieves more precise target boundary localization after resolution reconstruction. Precision remained stable at approximately 84%, suggesting that despite capturing more targets, the model did not experience a significant increase in false positive rates. This demonstrates that resolution reconstruction successfully optimizes the model’s ability to capture small targets and enhances feature representation while improving image quality.

With the sequential introduction of the Hybrid Global Multi-Head Attention mechanism (HG-MHA), the SC-BiFPN module, and the C2f-Ghost-Sobel module, the model exhibited continuous improvements in Recall and mAP, while Precision remained stable. The HG-MHA mechanism boosted Recall to 79.74% and further increased mAP to 83.16%, showing that global modeling, local edge enhancement, and dynamic channel weighting enable the model to capture key targets more effectively in complex backgrounds. The incorporation of SC-BiFPN further enhanced the multi-scale feature fusion capability, raising Recall to 80.27% and steadily improving mAP to 83.35%. This underscores the critical role of cross-layer connections in enhancing feature flow and target detection performance. Lastly, the lightweight design of the C2f-Ghost-Sobel module reduced parameters and FLOPs while maintaining stable performance. Although mAP experienced a slight decline (to 83.32%), the model’s efficiency improved significantly, demonstrating its superiority in edge feature enhancement and computational cost control.

Overall, through resolution reconstruction and YOLOv8 module improvements, the model’s performance in infrared image object detection was significantly enhanced. Resolution reconstruction greatly improved input image quality, optimizing the detection of small and edge targets. The improved modules, leveraging global and local modeling, feature fusion, and edge enhancement, enhanced the model’s adaptability to complex scenarios. Ultimately, the model achieved notable improvements in Recall and mAP while maintaining stable Precision, validating the effectiveness and robustness of the proposed improvements in practical applications. These advancements provide critical references for addressing the challenges of small target detection in infrared images and lay a solid foundation for future research in infrared target detection.

• Item-by-Item analysis

From the results of the ablation experiments, it is evident that the introduction of the HG-MHA mechanism significantly improved the model’s performance, with Recall and mAP increasing to 79.74% and 83.16%, respectively. The HG-MHA mechanism enhances the capture of long-distance dependencies through multi-head global modeling, strengthens edge and texture features using local attention, and dynamically focuses on important feature channels via the channel weighting mechanism. This design significantly boosts the model’s target detection capabilities in complex backgrounds and low-contrast scenarios.

**Fig 10:**
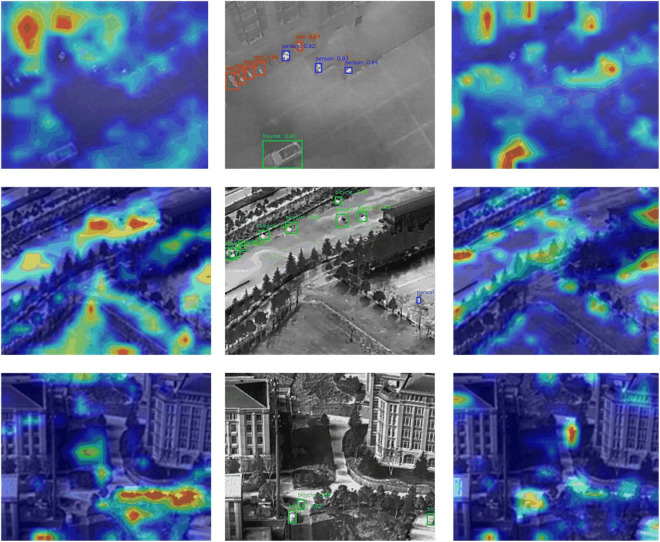
Attention Distribution Visualization. (a) Without Attention Mechanism; (b) Original Infrared Image and Targets; (c) With Attention Mechanism.

The attention heatmaps (Fig 10) clearly demonstrate the substantial improvements brought by the HG-MHA mechanism in infrared image target detection. Without the attention mechanism, the heatmaps show dispersed responses in target areas, with some small targets completely ignored, while background noise is overly prominent, making it challenging for the model to distinguish targets from the background accurately. In contrast, after introducing the HG-MHA mechanism, the high responses in target regions became more concentrated, especially in detecting small targets and suppressing complex backgrounds. The global multi-head attention mechanism effectively integrates long-distance dependencies, enhancing the perception of global semantic information. Meanwhile, local attention strengthens edge and detail features, significantly improving small target detection. Additionally, the channel weighting mechanism dynamically adjusts feature weights, allowing the model to focus more effectively on critical areas and significantly suppress background noise. These improvements highlight the robustness and adaptability of the model under complex scenes and low-contrast conditions, validating the effectiveness of the attention mechanism in infrared image target detection.

The improved SC-BiFPN demonstrates its superiority in multi-scale feature fusion. By introducing critical cross-layer connections, such as P3 → P5 and P5 → P3, the model efficiently leverages features from different levels, raising Recall to 80.27% and mAP to 83.35%. This cross-layer interaction mechanism effectively supplements the connection between high-level semantic information and low-level detail features while avoiding the redundant computations introduced by GFPN’s fully connected paths.

To achieve a lightweight design, the C2f-Ghost-Sobel module further optimized the computational efficiency of the model. The experimental results show that while mAP slightly decreased to 83.32%, the parameters and FLOPs were reduced from 3.5M and 9.5 GFLOPs to 3.2M and 8.6 GFLOPs, respectively. By replacing standard convolutions with Ghost Convolutions, the computational load was significantly reduced. Simultaneously, the Sobel operator effectively enhanced edge feature extraction, particularly excelling in detecting targets with blurred boundaries. This module captures edge information in infrared images with high efficiency while maintaining a balance between performance and computational cost.

In summary, the resolution reconstruction module addresses the low-resolution issues of infrared images, laying the foundation for improved model performance. The hybrid attention mechanism enhances feature representation, the improved SC-BiFPN boosts multi-scale feature fusion, and the C2f-Ghost-Sobel module achieves a balance between performance and computational efficiency through lightweight design. Together, these modules synergistically improve the robustness and adaptability of the enhanced model in infrared image target detection tasks.

#### 4.4.4. Algorithm comparison.

This section presents a comprehensive comparison between the proposed method and several representative object detection models. Both quantitative metrics and qualitative visualizations are used to demonstrate the superiority and robustness of our approach in infrared scenarios.

• Quantitative Comparison on Multiple Datasets

To evaluate the generalization ability of the proposed ISR-YOLOv8, we conduct experiments on four public infrared datasets: HIT-UAV, FLIR, KAIST, and DroneVehicle. These datasets cover a wide range of application scenes, including aerial surveillance, low-light city streets, pedestrian tracking, and vehicle monitoring from drone perspectives. Each dataset presents unique challenges in terms of object size, background complexity, and imaging modality.

We compare ISR-YOLOv8(our algrithm) with six popular detection models: Faster R-CNN, SSD, YOLOv5s, YOLOv7, YOLO-IR-Free and YOLO-DeepOC-IR. The mAP results across datasets are summarized in Table 4. The proposed method consistently achieves the best detection performance on all datasets. On HIT-UAV, ISR-YOLOv8 reaches 83.32% mAP, outperforming YOLO-IR-Free (82.08%) and YOLO-DeepOC-IR (82.71%). On FLIR, KAIST, and DroneVehicle, our method also shows strong im-provements in accuracy, especially for small or distant targets.

**Table 4:** mAP (%) of each dataset under different object detection algorithms.

Dataset	Faster R-CNN	SSD	YOLOv5s	YOLOv7	YOLO-IR-Free	YOLO-DeepOC-IR	ISR-YOLOv8
HIT-UAV	75.48	76.75	79.62	81.23	82.08	82.71	83.32
FLIR	71.63	72.52	74.38	76.54	76.92	77.28	78.84
KAIST	68.9	69.44	71.08	73.26	73.84	74.15	75.47
DroneVehicle	70.31	71.02	72.79	74.91	75.66	75.92	77.14

• Qualitative comparison on HIT-UAV and FLIR

**Fig 11:**
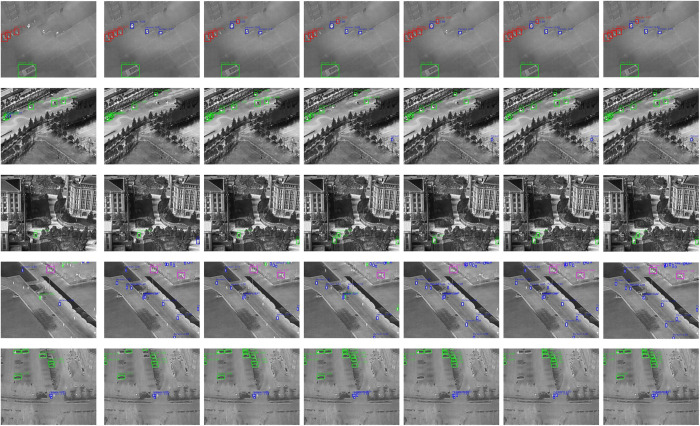
Detection results on the HIT-UAV dataset using different algorithms. From (a) to (g), the results correspond to Faster R-CNN, SSD, YOLOv5s, YOLOv7, YOLO-IR-Free, YOLO-DeepOC-IR, and ours, respectively.

**Fig 12:**
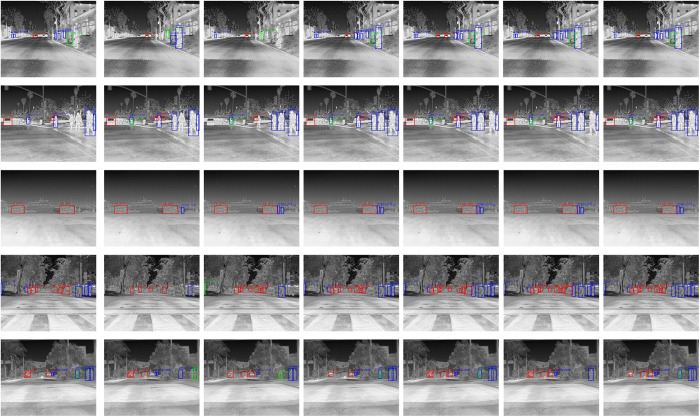
Detection results on the FLIR dataset using different algorithms. From (a) to (g), the results correspond to Faster R-CNN, SSD, YOLOv5s, YOLOv7, YOLO-IR-Free, YOLO-DeepOC-IR, and ours, respectively.

To visually demonstrate detection performance, we provide comparative visualizations on two representative datasets: HIT-UAV and FLIR. Detection results of seven different algorithms, including the proposed ISR-YOLOv8, are shown in Figs 11, 12.

On HIT-UAV, Faster R-CNN detects large objects effectively but fails on small and cluttered targets. SSD improves detection speed but exhibits poor boundary localization. YOLOv5s and YOLOv7 achieve more balanced detection but still generate false positives. YOLO-IR-Free and YOLO-DeepOC-IR, designed for infrared inputs, perform better in background suppression, yet their small object recall is limited. Our method, ISR-YOLOv8, outperforms all others by accurately identifying small, distant, or overlapping targets, while maintaining high precision and robustness in cluttered or low-contrast areas. The improvements come from its joint use of super-resolution recon-struction, hybrid attention design, SC-BiFPN structure, and the C2f-Ghost-Sobel module.

On the FLIR dataset, similar trends are observed. ISR-YOLOv8 accurately distinguishes objects under low-light and high-noise conditions. Compared with the other methods, it achieves clearer boundary delineation and fewer false alarms, particularly when detecting small pedestrians or vehicles in night-time city scenes. These visual comparisons strongly vali-date the effectiveness of our method in practical infrared scenarios.

## 5. Conclusion and limitation

In this study, we proposed ISR-YOLOv8, an improved object detection framework tailored for infrared imagery, incorporating advanced modules such as LightweightSRNet for super-resolution reconstruction, Hybrid Global Multi-Head Attention (HG-MHA) for feature enhancement, SC-BiFPN for multi-scale feature fusion, and C2f-Ghost-Sobel for lightweight edge feature extraction. The proposed approach effectively addresses the challenges posed by low-resolution, complex backgrounds, and indistinct boundaries in infrared images, as validated by experiments conducted on HIT-UAV, FLIR, KAIST and DroneVehicle datasets. The results demonstrated significant improvements in recall and mAP, while maintaining stable precision, confirming the robustness and efficacy of ISR-YOLOv8 in detecting small and challenging targets.

However, this work has certain limitations. First, while ISR-YOLOv8 achieves remarkable performance improvements, the added computational cost, though minimized, could still be challenging for real-time applications on extremely resource-constrained devices. Second, the current approach primarily focuses on single-frame infrared images and does not consider temporal information, which could further enhance detection performance in video sequences. Lastly, the adaptability of the model to different infrared imaging modalities and more diverse datasets remains an area for further exploration.

Future work will focus on optimizing the computational efficiency of the model for real-time embedded applications and extending the framework to leverage spatio-temporal information in sequential data. Additionally, we plan to explore the integration of domain adaptation techniques to improve the generalization of ISR-YOLOv8 across a broader range of infrared imaging scenarios. Despite these limitations, the proposed method provides a robust foundation for advancing infrared image-based object detection, especially in scenarios involving small and challenging targets.

## Supporting information

S1 Data Raw performance data for the first algorithm shown in Fig 9. (CSV)

S2 Data Raw performance data for the second algorithm shown in Fig 9. (CSV)

S3 Data Raw performance data for the third algorithm shown in Fig 9. (CSV)
